# Dietary Caffeine, Cold Exposure, and the Estrogen–TRPM8 Axis: A Nutri-Environmental Model for Lower Urinary Tract Symptoms in the Menopause Transition: A Narrative Review

**DOI:** 10.3390/nu18050825

**Published:** 2026-03-03

**Authors:** Dong Hee Lee, Jeong Jun Park

**Affiliations:** 1 Department of Obstetrics and Gynecology, Kangbuk Samsung Hospital, Sungkyunkwan University School of Medicine, Seoul 03181, Republic of Korea; wooahan.obgy@gmail.com; 2 Department of Anesthesiology and Pain Medicine, CHA Bundang Medical Centre, CHA University School of Medicine, Seongnam 13496, Republic of Korea

**Keywords:** caffeine pharmacokinetics, dietary triggers, water homeostasis, menopause transition, nocturia, cold exposure, TRPM8, copeptin

## Abstract

**Background/Objectives**: Lower urinary tract symptoms (LUTSs), particularly nocturia and urgency, often intensify during the menopause transition and may worsen with caffeine intake and cold exposure. This review aims to synthesize evidence relevant to a hypothesized caffeine–cold interaction in transitional menopause, focusing on water homeostasis and the estrogen–transient receptor potential melastatin 8 (TRPM8) cold-sensory axis, and to propose potentially actionable, nutrition-centered intervention candidates for future testing. **Methods**: Structured narrative review of PubMed, Embase, Web of Science, and citation tracking (inception–January 2026). Evidence was mapped into a mechanistic framework distinguishing established from hypothesis-generating links; no formal systematic-review study selection or meta-analysis was performed. **Results**: Caffeine can increase urine output via renal mechanisms (adenosine receptor antagonism and natriuresis) and may lower bladder sensory thresholds. Because half-life is long and variable, afternoon intake can extend into sleep, potentially increasing awakenings and nocturnal voids. Human studies link colder indoor environments to nocturia/overactive bladder, and passive pre-bedtime heating is associated with fewer nocturnal voids. We propose that repeated nighttime cold may amplify caffeine-related diuresis and may shift urine production toward the night, while estradiol decline may heighten TRPM8-mediated cold sensory gain, potentially contributing to urgency/frequency flares. A testable 2 × 2 cold × caffeine framework can operationalize dose, timing, and metabolism, pairing voiding diaries and bedroom temperature sensing with copeptin profiling. **Conclusions**: Transitional menopause may represent a susceptibility window in which endocrine instability and estradiol decline could plausibly increase sensitivity to indoor cold exposure and caffeine intake, potentially contributing to nocturia and urgency. The hypothesis label ‘dual hormone suppression’ (attenuated nocturnal AVP signal plus estradiol decline) may provide a mechanistic substrate for cold-exacerbated nocturnal polyuria, while an estrogen–TRPM8 axis may amplify cold-evoked urgency. Potentially actionable candidates include chronobiological caffeine timing/management and low-burden thermal strategies; nevertheless, menopause-stage-specific epidemiologic and clinical evidence for a caffeine × cold interaction remains limited and several mechanistic links are extrapolated, so prospective diary- and biomarker-enabled studies and controlled trials are needed to validate mechanisms and refine cold-sensitive endotypes.

## 1. Introduction

The menopause transition is characterized by fluctuating ovarian function and progressive estrogen decline and can be staged using the Stages of Reproductive Aging Workshop + 10 (STRAW + 10) criteria [1]. Beyond vasomotor symptoms, menopause-related metabolic, vascular, immune, and neural changes may influence pelvic organ function [2], and cohort data indicate meaningful midlife health transitions across this window [3].

Lower urinary tract symptoms (LUTSs; urgency, frequency, nocturia, and urinary incontinence) are common in midlife women. Longitudinal studies show increasing risk of incident urinary incontinence across menopausal transition stages [4], and storage symptoms can emerge during early transition [5]. Recent cohort work further supports stage-related changes in bladder health, motivating phenotype- and stage-specific approaches [6].

Focusing on the menopause transition (rather than established postmenopause) is clinically relevant because LUTSs may emerge or accelerate during this dynamic window of endocrine instability and sleep vulnerability, creating an opportunity for early, low-risk prevention. Cold-induced urinary urgency is frequently reported by patients; transient receptor potential melastatin 8 (TRPM8) is a cold-sensitive channel proposed to contribute to cold-evoked urgency, and estradiol decline may plausibly heighten cold sensory gain along this pathway. In this review, we use phenotype to denote symptom patterns, endotype to denote putative mechanistic drivers, and exposure-defined subgroup for pragmatic stratification based on caffeine dose/timing and indoor temperature.

Guidelines and reviews often emphasize postmenopausal genitourinary syndrome and chronic overactive bladder, with less focus on transitional menopause and seasonally varying triggers such as indoor cold or caffeine dose/timing. The genitourinary syndrome framework and North American Menopause Society position statement guide staged assessment and local estrogen therapy [7,8], and overactive bladder guidelines outline evidence-based tiers [9]. However, indoor temperature, cold sensory mechanisms, and mechanistic phenotyping for nocturia are rarely integrated, despite standardized nocturia terminology [10]. Nutrition guidance also tends to treat caffeine qualitatively rather than as a dose- and time-dependent exposure.

Rising household energy costs have intensified energy poverty and contributed to colder homes during winter [11]. Energy poverty is linked to adverse health outcomes and excess winter mortality in the European Union, with disproportionate impacts on women and vulnerable households [12,13]. These trends may increase exposure to nighttime indoor cold and could plausibly exacerbate cold-sensitive LUTSs, making low-burden environmental strategies a potentially relevant public health approach.

**Figure 1:**
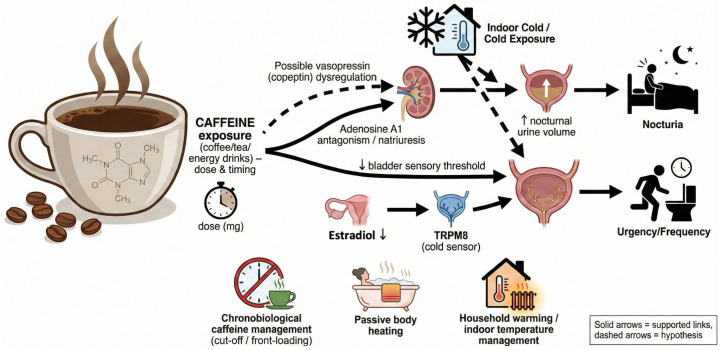
Proposed nutri-environmental model linking caffeine exposure, indoor/bedroom cold, and estradiol decline to cold-sensitive lower urinary tract symptoms (LUTSs) during the menopause transition. Solid arrows denote supported pathways; dashed arrows denote hypothesis-generating links. The intervention panel highlights earlier-day caffeine and low-burden thermal strategies. Abbreviations: LUTSs, lower urinary tract symptoms; TRPM8, transient receptor potential melastatin 8.

Cold exposure and caffeine intake are common, measurable exposures that vary by season and daily routine. Patients commonly report winter worsening, cold-induced urgency, and caffeine-associated nocturia flares, suggesting a cold-sensitive LUTS pattern. Caffeine clearance varies widely, largely via cytochrome P450 1A2 (CYP1A2), and because adult half-life is long and variable, later-day intake can extend into sleep [14]. Here, we propose a nutri-environmental framework in which recurrent indoor/bedroom cold and caffeine exposure (dose and timing) may interact with estradiol decline to influence nocturnal water homeostasis and cold-evoked urgency (Figure 1). Given the scarcity of menopause-stage-stratified epidemiologic/clinical data on cold × caffeine interactions, several mechanistic links are extrapolated and presented explicitly as hypothesis-generating.

Objectives and central hypothesis: Because LUTS triggers during the menopause transition remain understudied and menopause-stage-stratified evidence on cold exposure and caffeine intake is scarce, we conducted a structured narrative review to (1) synthesize clinical and mechanistic evidence relevant to cold exposure and caffeine as modifiable triggers of LUTSs during the menopause transition, (2) integrate water-homeostasis pathways (AVP/copeptin–V2R/AQP2) with an estrogen-modulated cold-sensory pathway (TRPM8), and (3) propose a testable cold × caffeine framework with measurable exposures and pragmatic, nutrition-centered interventions. We hypothesize that estradiol decline and endocrine instability could heighten TRPM8-mediated cold sensory gain and reduce overnight concentrating reserve, such that repeated indoor/bedroom cold may amplify caffeine-related nocturnal urine production and urgency/frequency flares. This hypothesis is intended to motivate prospective validation rather than to imply established causal links, and it can be operationalized using frequency–volume charts, time-stamped caffeine logs, and bedroom temperature recording.

Caffeine is a measurable exposure with substantial inter-individual pharmacokinetic variability; accordingly, we emphasize time-stamped dose and timing (rather than beverage labels alone) for both clinical counseling and future trials.

## 2. Materials and Methods

We conducted a structured narrative review and hypothesis-generating synthesis to map clinical and experimental evidence onto an integrative model. This work was not designed as a systematic review and therefore did not apply formal systematic-review study selection or meta-analysis. We searched PubMed, Embase, and Web of Science from inception through January 2026 and performed citation tracking of key articles. We combined controlled vocabulary terms and free text keywords for menopause transition, perimenopause, nocturia, nocturnal polyuria, overactive bladder, indoor temperature, cold exposure, vasopressin, copeptin, TRPM8, sleep, falls, caffeine, and energy poverty. We prioritized peer-reviewed human studies in midlife women (operationally defined as approximately 40–60 years and/or studies reporting STRAW + 10 transition stages when available) that reported urinary outcomes and specified menopausal stage or a relevant age window. When evidence in midlife women was limited, we included relevant older adult human studies to inform exposure response hypotheses. We also included translational studies that examined vasopressin-dependent cold diuresis or TRPM8-mediated cold urgency to inform mechanistic inference. We extracted study design, population characteristics, exposure definitions, urinary endpoints, and mechanistic readouts. We synthesized evidence narratively and integrated findings into a framework that separates established evidence from hypothesis-generating links. Representative database-specific query strings and the qualitative evidence-grading rubric used to distinguish human interventional, human observational, mechanistic physiology, animal/ex vivo, and hypothesis-only links are provided in Supplementary Table S1.

## 3. Results

### 3.1. Epidemiology and Clinical Patterns of Lower Urinary Tract Symptoms Across the Menopause Transition

#### 3.1.1. Symptom Emergence During Transition

Longitudinal cohorts show that urinary incontinence incidence increases across menopausal transition stages [4]. Early work also reports rising bladder symptoms during the early transition [5]. Recent midlife cohort data further link menopausal status and hormone use with bladder health and LUTS, reinforcing transitional menopause as a distinct phenotyping window [6].

#### 3.1.2. Overlap with Genitourinary Syndrome of Menopause and Overactive Bladder

Genitourinary syndrome of menopause provides a framework for estrogen-deficiency-related urinary symptoms and supports targeted local estrogen therapy [7,8]. However, it does not fully explain cold-triggered urgency or nocturia surges. Overactive bladder guidelines emphasize behavioral therapy and pharmacologic escalation but do not explicitly integrate indoor cold exposure or cold sensory mechanisms [9].

### 3.2. Cold Exposure and Indoor Temperature as Modifiable Determinants of Nocturia and Urgency

#### 3.2.1. Seasonality and Symptom Worsening

Seasonal variation in overactive bladder symptoms has been reported in female patient cohorts, consistent with winter worsening in storage symptoms [15]. These patterns align with patient narratives of cold-induced urgency and nocturia flares.

#### 3.2.2. Indoor Cold Exposure and Nocturia

Community and nationwide studies report higher nocturia and overactive bladder symptom probability in colder indoor environments [16,17], supporting indoor temperature as a measurable, modifiable exposure.

#### 3.2.3. Passive Body Heating as a Behavioral Countermeasure

Passive body heating before bedtime has been associated with fewer nocturnal voids during the cold season in older adults [18]. Although generalizability to transitional menopause is uncertain and stage-stratified data in midlife women are limited, this finding motivates pragmatic trials that quantify bedroom temperature and test low-burden warming strategies alongside diary-based urinary outcomes. Representative human evidence on seasonality, indoor cold exposure, and passive body heating is summarized in Table 1.

### 3.3. Mechanistic Synthesis I: A Hypothesis-Generating Dual-Axis Model for Cold-Exacerbated Nocturnal Polyuria

#### 3.3.1. Cold-Induced Diuresis and Vasopressin Dependence

Cold exposure can induce diuresis. In rats, genetic AVP deficiency abolishes cold-induced diuresis, implicating an intact vasopressin axis [19], and additional work supports antidiuretic hormone involvement in cold diuresis [20]. Classic laboratory cold diuresis is an acute response to peripheral vasoconstriction and central blood volume shift; in contrast, wintertime indoor/bedroom cold represents a recurrent (often nightly) stressor. We hypothesize that in susceptible states—where the nocturnal AVP rise is blunted and/or renal responsiveness is reduced—even modest cold exposure may shift urine production toward the sleep period and contribute to persistent nocturnal polyuria. These findings should be interpreted as mechanistic evidence that an intact vasopressin axis is required to generate the acute cold-diuretic phenotype in experimental settings [19,20], rather than as evidence that vasopressin deficiency explains nocturnal polyuria. Our central hypothesis operates at a different level: during the menopause transition, attenuation of the nocturnal vasopressin rhythm and/or collecting duct responsiveness may reduce overnight concentrating reserve. Under repeated indoor or bedroom cold exposure, this reduced reserve may shift a greater fraction of 24-h urine production into the sleep period, increasing nocturnal urine volume and symptom penetrance without implying absolute vasopressin deficiency.

#### 3.3.2. Caffeine as a Core Dietary Determinant of Water Homeostasis and Bladder Sensitivity

Coffee intake has been linked to vasopressin-system readouts, including copeptin, in epidemiologic and experimental work [21]. Beyond beverage volume, caffeine promotes diuresis and natriuresis via renal mechanisms (including adenosine A1 receptor antagonism) [22] and can worsen bladder function parameters in patients with overactive bladder symptoms [23]. Systematic review evidence suggests that targeted caffeine reduction can improve urgency and frequency, although effect sizes vary and adherence is challenging [24].

For nutrition-centered translation, caffeine should be operationalized by source-specific content, mg dose, and timing relative to bedtime, with attention to metabolic variability. Adult caffeine half-life is long and variable (commonly 3–7 h), so afternoon intake can extend into the sleep period and increase awakenings [14]. Simple phenotyping using habitual intake patterns and urinary caffeine metabolites (e.g., paraxanthine) may help explain heterogeneous urinary responses. Supplementary Table S2 provides the approximate caffeine content across common sources to support feasible stepwise tapering and substitution. From an exposure-science perspective, separating caffeine from co-ingested fluid volume is essential because the same mg dose can be delivered by coffee, tea, energy drinks, or supplements. Very high habitual intake has been associated with urgency urinary incontinence risk in women (e.g., ≥450 mg/day) [25], and experimental challenge studies in adults with overactive bladder symptoms report earlier urgency and diuresis after ~4.5 mg/kg caffeine [23]. Together, these data support time- and dose-focused, individualized ‘front-loading’ rather than blanket prohibition, with outcomes tracked using nocturnal urine volume and sleep continuity [24].

#### 3.3.3. Menopause Transition as a Susceptibility State

The menopause transition may amplify vulnerability to nocturnal polyuria through altered neuroendocrine regulation, sleep/circadian disruption, and reduced renal concentrating reserve. Endocrine instability and estradiol decline may plausibly modulate the nocturnal AVP rhythm and/or collecting-duct responsiveness, although stage-stratified human data remain sparse. Experimental work shows age-associated downregulation of renal vasopressin V2 receptor (V2R) and aquaporin-2 (AQP2) expression paralleling defects in urine concentration [26], and AQP2 responds dynamically to changes in vasopressin signaling and hydration state [27]. While human cold-specific renal adaptations are incompletely characterized, these findings support a ‘limited reserve’ context in which cold and caffeine perturbations more readily translate into nocturnal urine production.

#### 3.3.4. Proposed Model: Dual Hormone Suppression

In this review, we use the term “dual hormone suppression” as a conceptual shorthand for a candidate susceptibility state, and not as a claim of documented endocrine suppression in transitional menopause. Here, dual hormone suppression denotes a susceptibility state defined by the attenuation of the nocturnal AVP signal together with progressive estradiol decline during the menopause transition; estradiol decline is also hypothesized to heighten TRPM8-mediated cold sensory gain, linking nocturnal polyuria and urgency pathways within a single framework. Caffeine is treated as an exposure (not a hormone): it can increase urine output via vasopressin-independent tubular and hemodynamic mechanisms [22] and may influence nocturnal vasopressin dynamics through circadian disruption and sleep fragmentation, although direct causal evidence in midlife women is limited [21]. Cold stress perturbs vasopressin-dependent diuresis in experimental models [19,20]. We hypothesize that estradiol decline modifies both renal concentrating reserve and TRPM8-mediated cold sensory gain, yielding nocturnal polyuria- and urgency-predominant phenotypes that can be tested in controlled cold × caffeine studies.

In randomized crossover studies, afternoon caffeine may increase nocturnal urine volume and alter nocturnal copeptin profiles versus placebo, with effect modification by habitual intake and caffeine metabolism. We propose nocturnal urine volume as the primary endpoint, with void frequency and sleep fragmentation prespecified as secondary outcomes.

1. Cold exposure may amplify the nocturnal urine-volume response to caffeine under controlled hydration, beyond additive effects;
2. Pre-sleep thermal interventions may attenuate nocturnal urine volume and nocturnal voids during cold exposure, with larger effects in cold-sensitive phenotypes.

These predictions are testable in prospective cohorts and randomized crossover trials that manipulate caffeine timing and thermal exposure.

### 3.4. TRPM8 and Cold-Induced Urinary Urgency

#### 3.4.1. Role of TRPM8 and Cold-Induced Urinary Urgency

TRPM8 is a cold-sensitive channel implicated in cold-evoked urinary urgency. In an animal model of acute cold-induced urinary urgency, TRPM8 is essential for acute cold-induced urgency [28]. In ovariectomized rat models, cold stress-induced detrusor overactivity involves alpha1-adrenergic pathways and TRPM8-related mechanisms, supporting estrogen-deficiency sensitivity [29].

#### 3.4.2. TRPM8 Expression in Bladder Disorders and Therapeutic Antagonism

TRPM8 expression has been described in human urinary bladder disorders with clinical correlations [30]. Pharmacologic TRPM8 blockade reduces bladder reflex activity in rodent models of overactive and painful bladder syndromes [31], and structural studies clarify ligand and lipid sensing that may enable rational antagonist development [32].

#### 3.4.3. Estrogen Modulation of TRPM8 and Sensory Gain

Estrogen deficiency may enhance TRPM8-related sensory signaling and lower the threshold for cold-induced urgency. Ovariectomized models suggest that beta-estradiol can modulate TRPM8 expression or function in cold-sensitive contexts [33], but direct human evidence linking estradiol dynamics to TRPM8-mediated bladder sensation is limited. Serial estradiol profiling combined with standardized cold challenge testing is needed to validate this axis.

#### 3.4.4. Integrated Clinical Hypothesis

We propose that transitional menopause predisposes to cold-induced urgency through the convergence of TRPM8-mediated afferent activation and sympathetic amplification. Additional contributors such as urothelial remodeling or hypoxia-related signaling have indirect support and should be treated as hypothesis-generating [34].

## 4. Discussion

This review integrates epidemiologic, mechanistic, and environmental evidence into a nutri-environmental model of cold-sensitive LUTS during the menopause transition, emphasizing a hypothesis-generating dual-axis model (attenuated nocturnal AVP signaling plus estradiol decline) and a candidate estrogen–TRPM8 sensory axis.

Indoor temperature is a biologically active exposure: colder homes are associated with nocturia and overactive bladder symptoms [16,17], and passive pre-bedtime heating is linked to fewer nocturnal voids in cold seasons [18]. Future work should treat bedroom temperature as a time-varying exposure and test pragmatic warming strategies alongside diary-based outcomes.

Experimental evidence supports vasopressin dependence of cold diuresis [19,20], and age-related downregulation of V2R/AQP2 pathways may reduce concentrating reserve [26]. These mechanisms support phenotyping nocturnal polyuria using nocturnal urine volume, serum/urine osmolality, and copeptin in midlife women.

Within this model, caffeine may act through both renal diuretic/natriuretic pathways and sleep-mediated mechanisms. Human data link coffee/caffeine exposure to vasopressin-system readouts [21] and renal physiology supports caffeine-induced diuresis [22]. Direct evidence for a caffeine × cold interaction in transitional menopause is limited; the key contribution is a falsifiable framework for mechanistic and pragmatic trials.

TRPM8 provides a plausible sensory mediator of cold-evoked urgency: TRPM8 is required for acute cold-induced urgency in experimental models [28], and ovariectomy/cold-stress data implicate sympathetic pathways and TRPM8 upregulation [29,33]. Human bladder expression studies support clinical relevance [30], and preclinical antagonist work plus structural biology highlight druggability [31,32]. Human stage-specific validation remains a priority.

Existing evaluation tools—frequency–volume charts and standardized nocturia terminology—are well-suited to incorporate time-stamped caffeine intake and objective bedroom temperature sensing [10,35]. This enables exposure-based phenotyping and low-risk, scalable lifestyle trials before pharmacologic escalation. In trials, separating nocturnal urine volume from void frequency is critical, because sleep fragmentation can increase nocturia without true nocturnal polyuria; pairing frequency–volume charts with sleep diaries or actigraphy improves attribution [36,37]. Because nocturia and insomnia can reinforce each other via sleep fragmentation, assessment should explicitly consider the directionality of awakenings and voids. Nocturia can disrupt sleep continuity, while insomnia or other sleep disorders can increase ‘opportunistic’ nocturnal voiding that mimics nocturnal polyuria. Accordingly, we recommend pairing frequency–volume charts (including nocturnal urine volume) with sleep diaries or actigraphy to determine whether awakenings precede voiding, and screening for common sleep comorbidities when nocturia appears disproportionate to nocturnal urine volume.

Biomarker endotyping is promising but preliminary. Urinary microbiome studies differentiate women with urgency urinary incontinence and show increased urinary Lactobacillus with vaginal estrogen therapy [38,39]. Urinary metabolomics may index overactive bladder severity and could quantify caffeine metabolites [40]. Renal concentrating physiology can be profiled using urine osmolality, sodium/urea excretion, and exploratory urinary AQP2 measures, alongside copeptin [21,26,27]. Future studies could explore urine-derived extracellular vesicles as a low-burden source of collecting-duct markers, such as extracellular vesicle aquaporin-2 (EV-AQP2), and sensory-channel transcripts, alongside electrolytes and osmolality.

Because nocturia and insomnia often coexist, integrated cognitive behavioral strategies may complement exposure modification [41]. Nocturia fragments sleep and is associated with daytime impairment. In older and community-dwelling populations, nocturia is linked to increased falls and fractures [42,43]; while midlife data are limited, wintertime nocturia combined with nighttime ambulation raises plausible safety concerns.

### 4.1. Future Directions

Central hypothesis: Transitional menopause may be a susceptibility window in which indoor cold and caffeine exposure may interact with AVP-related water homeostasis and an estrogen–TRPM8 sensory axis, potentially worsening nocturia and urgency. Prospective cohorts should combine STRAW + 10 staging with objective indoor temperature sensing, time-stamped caffeine intake, and frequency–volume charts to test key predictions and quantify effect modification. Given that several mechanistic links are extrapolated, Table 2 is intended as a measurement roadmap for falsifiable testing in midlife women rather than a claim of established causality.

**Figure 2:**
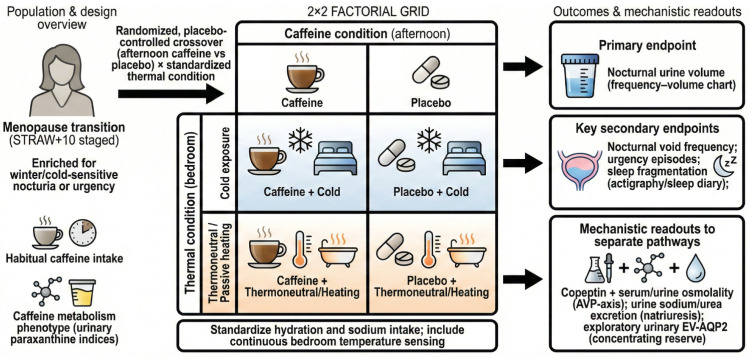
Proposed 2 × 2 cold × caffeine framework for mechanistic and pragmatic testing in the menopause transition. Primary outcome: nocturnal urine volume; secondary outcomes: nocturnal voids, urgency, and sleep fragmentation; mechanistic readouts include copeptin and osmolality (plus urinary sodium/urea and exploratory EV-AQP2). Abbreviations: AQP2, aquaporin-2; AVP, arginine vasopressin; EV-AQP2, extracellular vesicle aquaporin-2; STRAW + 10, Stages of Reproductive Aging Workshop + 10.

Controlled mechanistic studies are needed. A randomized crossover trial can compare afternoon caffeine versus placebo under standardized thermal conditions, with nocturnal urine volume and copeptin dynamics as primary mechanistic outcomes. Pragmatic trials can test passive body heating or bedroom warming during cold seasons, with nocturia, sleep continuity, and falls prespecified where feasible [18,37,42,43]. Figure 2 summarizes a 2 × 2 cold × caffeine framework, and Table 2 maps mechanisms to measurements. In the 2 × 2 framework, participants can be enriched for self-reported cold-sensitive nocturia/urgency and stratified by habitual caffeine intake and a simple metabolism proxy (e.g., urinary paraxanthine ratios). Continuous bedroom temperature sensing and time-stamped beverage logs can verify exposure fidelity, while standardized evening fluid and sodium intake reduces confounding. Pre-specifying renal (nocturnal urine volume, sodium excretion) versus sensory (urgency episodes) endpoints can help separate diuretic from afferent pathways.

Prioritized research questions include: (1) Do colder bedroom temperatures shift nocturnal urine volume and nocturia risk in stage-stratified midlife cohorts? (2) Does afternoon caffeine increase nocturnal urine volume and sleep fragmentation, and is this effect modified by cold exposure under standardized hydration? (3) Do copeptin dynamics (and urine osmolality/sodium) differ by menopausal stage or estradiol variability during cold exposure? (4) Can passive body heating/bedroom warming reduce nocturnal urine volume, urgency, and awakenings in cold-sensitive symptom patterns? (5) Does estradiol decline predict heightened cold-evoked urgency responses consistent with TRPM8-mediated sensory gain?

To validate the estrogen–TRPM8 axis, studies should pair STRAW + 10 staging and serial estradiol measures with objective indoor temperature sensing and standardized cold-challenge protocols, testing whether estradiol decline and TRPM8-related readouts predict larger cold-evoked urgency responses [28,29,30,31,32,33]. For exposure quantification, urinary caffeine metabolites can complement time-stamped intake records. Candidate panels for renal concentrating physiology include copeptin, serum/urine osmolality, urine sodium/urea, and exploratory urinary AQP2 measures [21,26,27]. Multi-omics (microbiome, metabolomics) may refine cold-sensitive endotypes [38,39,40].

### 4.2. Clinical Implications: Mechanism-Based Precision Lifestyle Medicine and Seasonal Tailoring

To translate the proposed cold × caffeine framework into practice, we outline a pragmatic assessment and seasonally tailored intervention approach. From a nutrition perspective, the clinically actionable unit is not ‘coffee’ per se but caffeine exposure defined by source, dose in milligrams, and timing relative to sleep, alongside substitution and tapering strategies that preserve adherence. This exposure science framing enables measurable interventions that are aligned with Nutrients readership: time-stamped intake logs, estimation of total daily caffeine load, and when feasible, metabolite-based phenotyping to account for inter-individual variability in clearance. Embedding these parameters into symptom diaries and frequency–volume charts supports mechanism-linked outcomes such as nocturnal urine volume and sleep continuity, and converts lifestyle advice into testable, nutrition-centered prescriptions.

#### 4.2.1. Symptom-Pattern and Mechanism-First Assessment

Clinical evaluation should differentiate nocturnal polyuria, reduced bladder capacity, sleep-driven awakenings, or mixed etiologies using frequency–volume charts and history [10,36], and assess genitourinary syndrome where relevant [7,8].

#### 4.2.2. Pragmatic Clinical Algorithm for Winter/Cold-Sensitive LUTS (For Implementation and for Future Trials)

Capture the symptom pattern and exposure profile: 3-day frequency–volume chart plus time-stamped caffeine intake and indoor/bedroom temperature (or proxy measures);

Classify mechanism (nocturnal polyuria vs. reduced bladder capacity vs. sleep-driven awakenings vs. mixed) to match interventions and outcomes, and assess concurrent insomnia/sleep disorders when awakenings appear disproportionate to nocturnal urine volume;

Trial low-burden measures: front-load caffeine earlier and avoid/reduce after ~14:00; taper dose stepwise if needed; pair with passive body heating and/or bedroom warming during cold periods while keeping evening fluids/sodium stable;

Reassess after 2–4 weeks; if nocturnal polyuria persists, consider AVP-related phenotyping (copeptin, serum/urine osmolality) and guideline-based escalation as indicated.

Implementation note: translate mg targets using Supplementary Table S2 and taper gradually (e.g., ~25–50 mg every 3–4 days) to limit withdrawal. Track nocturnal urine volume and sleep continuity, not voids alone. Where possible, substitute to lower-caffeine products after lunch (e.g., half-caff, tea, or decaf) rather than abrupt cessation, and document withdrawal symptoms (headache, fatigue) that may affect adherence. For phenotyping, record beverage type, serving size, and intake time, and keep total evening fluid volume stable when testing timing effects.

#### 4.2.3. Environmental Prescription and Heat-Based Interventions

Indoor temperature management is a modifiable exposure domain. Observational evidence links colder indoor temperatures to nocturia and overactive bladder symptoms [16,17], and passive body heating before sleep may reduce nocturnal voids during cold seasons [18]. These low-risk strategies are suitable for pragmatic ‘environmental prescription’ trials.

#### 4.2.4. Chronobiological Caffeine Management

Caffeine restriction is commonly recommended, and observational data link higher caffeine intake with urinary incontinence risk in women [25]. A nutrition-centered approach may improve feasibility: rather than universal elimination, align caffeine with chronobiology by concentrating intake earlier in the day and avoiding/reducing after ~14:00 to account for typical adult half-life [14]. During cold seasons or on colder indoor days, stepwise dose reduction or switching to lower-caffeine/decaffeinated options after lunch may help reduce nocturnal polyuria, urgency, and sleep fragmentation while aiming to preserve daytime function, although direct menopause-stage-specific evidence remains limited.

#### 4.2.5. Pharmacologic Strategies and Future Seasonal Sensory Targeting

Guideline-based overactive bladder therapies (antimuscarinics, beta-3 agonists, and escalation pathways) remain appropriate [9], and local estrogen is foundational when genitourinary syndrome is present [8]. For nocturnal polyuria, desmopressin can be effective but requires careful patient selection and serum sodium monitoring to mitigate hyponatremia risk [44,45]; meta-analytic evidence in women supports efficacy with safety frameworks [46].

Seasonal sensory pharmacology is emerging. TRPM8 antagonism shows preclinical activity and has been proposed as a therapeutic target for bladder disorders [30,31,32]. In women with cold-induced urgency, especially those with estrogen deficiency features, seasonal trials of sensory targeting combined with local estrogen are a rational future direction.

#### 4.2.6. Sleep, Falls, and Behavioral Interventions

Nocturia and insomnia can reinforce each other via sleep fragmentation [37]. Integrated cognitive behavioral approaches may reduce treatment burden when both are present [41]. Given associations of nocturia with falls and fractures in older adults [42,43], winter trials should also consider safety-focused counseling (e.g., night lighting and fall prevention) where relevant.

### 4.3. Research Gaps and Priorities

1. Human validation of TRPM8 linked cold urgency in transitional menopause, including quantitative mapping of cold sensory thresholds and bladder reflex responses;
2. Prospective studies integrating indoor temperature sensing, caffeine timing, copeptin profiles, and voiding diaries to test dual hormone suppression;
3. Biomarker driven phenotyping to define cold-sensitive endotypes including urinary microbiome and metabolomics signatures;
4. Intervention trials of environmental prescription, passive body heating, and chronobiological caffeine timing, stratified by menopausal stage and symptom mechanism.

### 4.4. Limitations

Perimenopause is heterogeneous and many studies use self-report or broad categories rather than STRAW + 10 staging, risking misclassification and diluted stage-specific associations [1,3,4,5,6]. Most human studies of seasonality and indoor temperature are not stratified by menopausal stage, so relevance to the menopause transition is inferred rather than directly tested. Evidence for caffeine-driven suppression of nocturnal vasopressin in peri/postmenopausal women is limited; confounding by fluid intake, comorbidity, and sleep disruption remains plausible [21,22,23,24]. The estrogen–TRPM8 axis is supported mainly by animal and tissue studies, with sparse human data directly linking estradiol dynamics to cold-evoked urgency [28,29,30,31,32,33]. As a hypothesis-generating review, we do not provide pooled effect estimates; heterogeneity in definitions and measurement may bias apparent consistency [10,35,36].

Interpretation is influenced by study availability and by the narrative-review design; although we used a structured search, we did not conduct formal systematic-review study selection or meta-analysis, and residual selection bias is possible. Accordingly, several links in the proposed model reflect extrapolated mechanisms rather than direct epidemiologic/clinical associations in transitional menopause.

We therefore present the framework as hypothesis-generating and provide a mechanism-to-measurement map to enable stage-stratified replication, falsification, and refinement in prospective cohorts and controlled cold × caffeine trials. Finally, terms such as “dual hormone suppression” are used as hypothesis labels to organize testable predictions and should not be interpreted as established clinical entities.

## 5. Conclusions

Transitional menopause may represent a susceptibility window in which endocrine instability and estradiol decline could plausibly increase sensitivity to indoor cold exposure and caffeine intake, potentially contributing to nocturia and urgency. Dual hormone suppression (attenuated nocturnal AVP signal plus estradiol decline) may provide a mechanistic substrate for cold-exacerbated nocturnal polyuria, while an estrogen–TRPM8 axis may amplify cold-evoked urgency. Potentially actionable strategies may include chronobiological caffeine management and low-burden thermal interventions; however, direct menopause-stage-specific epidemiologic and clinical evidence for a caffeine × cold interaction remains limited and several mechanistic links are extrapolated, so prospective diary- and biomarker-enabled studies and controlled trials are needed to validate mechanisms and refine cold-sensitive endotypes.

## Figures and Tables

**Table 1:** Representative human evidence linking seasonality/indoor temperature and passive pre-bedtime heating to nocturia and overactive bladder symptoms.

Evidence Domain	Study (Ref)	Design/Population (as Described in Manuscript)	Exposure Definition	Urinary Outcome(s)	Key Take-Home Message for This Review
Seasonality	[15]	Female patient cohort	Season (winter vs. other seasons)	OAB symptom burden	OAB storage symptoms show seasonal variation consistent with winter worsening.
Indoor cold exposure	[16]	Cross-sectional community cohort	Colder indoor environment/indoor cold exposure	Nocturia	Colder indoor exposure is associated with higher nocturia probability.
Indoor temperature	[17]	Nationwide epidemiological study (Japan)	Cold indoor temperatures	OAB outcomes	Lower indoor temperature is associated with higher probability of OAB outcomes.
Passive body heating	[18]	Observational study during cold season	Passive body heating before bedtime	Nocturia	Passive body heating before sleep is associated with fewer nocturnal voids during cold seasons.

Abbreviations: OAB, overactive bladder. Associations are observational unless otherwise specified.

**Table 2:** Mechanism-to-measurement map for cold-exacerbated LUTSs during the menopause transition (indoor cold exposure and nocturnal water homeostasis, caffeine exposure parameters, and the estrogen–TRPM8 sensory axis).

Framework Axis	Pop/ Stage Tag	Evidence Status (in This Review)	Key Pathway (Simplified)	Predicted LUTS Phenotype	Candidate Measurements/Biomarkers	Mechanism-Aligned Levers	Representative Refs
Caffeine exposure parameters (dose and timing)	G	Supported for acute bladder effects and epidemiologic risk at very high intake; timing-focused trials are limited [Evidence tag: L1–L2 for caffeine reduction/bladder outcomes; L3 for renal physiology; L5 for cold × caffeine synergy in transitional menopause]	Higher caffeine dose and later-day intake may increase urine output through mild diuresis and natriuresis and may worsen sleep continuity. In susceptible individuals, this could lower perceived bladder filling thresholds, contributing to urgency and frequency. Observational data suggest higher urgency urinary incontinence risk mainly at very high daily caffeine intake (for example, 450 mg per day or more), while experimental challenge studies report increased diuresis and earlier urgency at approximately 4.5 mg per kg in adults with overactive bladder symptoms	Possible dose- and timing-related nocturia and urgency flares. Risk may be higher with afternoon or evening caffeine, particularly when other winter triggers coexist	Daily caffeine dose (mg/day); timing relative to bedtime; beverage type; habitual intake; symptom/sleep diary; urinary caffeine metabolites (paraxanthine/theobromine/theophylline); exploratory CYP1A2 phenotyping	Chronobiological caffeine restriction (front-load earlier in the day; consider avoiding after 14:00); stepwise dose reduction or taper; switch to low-caffeine or decaffeinated beverages after lunch; combine with thermal strategies in winter	[14,23,24,25]
Estrogen–TRPM8 sensory axis	X + G	Supported in models; human stage-specific linkage is hypothesis-generating [Evidence tag: L4 for ovariectomy/animal models; L3 for mechanistic inference; L5 for stage-specific human validation]	Estrogen decline and endocrine instability may increase TRPM8 sensory gain; cold exposure triggers TRPM8 afferents with sympathetic amplification, leading to urgency reflex amplification	Cold-induced urgency and frequency flares; OAB-like storage symptoms with winter worsening	Symptom diary linked to temperature; OAB questionnaires; menopausal staging with serial estradiol profiling; cold sensitivity testing; standardized cold challenge response; exploratory TRPM8 assays in urine or tissue	Warming strategies; local estrogen when indicated; guideline-based OAB therapy; future seasonal trials of TRPM8 antagonism	[15,17,28,29,30,31,32,33]
Downstream sleep and safety outcomes	O + G	Supported [Evidence tag: L1–L2 for behavioral sleep interventions and risk associations]	Nocturia → sleep fragmentation → daytime impairment → falls/fractures risk	Sleep disruption with increased fall risk	Sleep diary/actigraphy; nocturnal awakenings; fall events; fracture outcomes	Integrated cognitive behavioral therapy (CBT) for nocturia + insomnia; nighttime lighting; fall-prevention counseling; treat upstream nocturia triggers	[37,41,42,43]
Indoor cold exposure and nocturnal water homeostasis (AVP/copeptin–V2R/AQP2)	G + O + X	Supported for indoor temperature associations with nocturia/OAB and for passive heating associations during cold seasons; supported for vasopressin dependence of cold diuresis and for V2R/AQP2 “concentrating reserve” mechanisms largely in experimental models. Transitional-menopause stage–specific nocturnal AVP attenuation/limited-reserve inference remains hypothesis-generating	Repeated nocturnal indoor/bedroom cold → cold stress (vasoconstriction/central volume shift) → diuresis with intact AVP-axis involvement; in a “limited reserve” state (blunted nocturnal AVP rise and/or reduced renal V2R/AQP2 responsiveness) → reduced collecting-duct water reabsorption → increased nocturnal urine volume (nocturnal polyuria) → nocturia	Cold-exacerbated nocturnal polyuria–predominant nocturia (higher nocturnal urine volume and nocturnal voids), with symptom improvement expected under thermoneutral bedroom/passive heating conditions	Objective bedroom temperature sensing (continuous/time-stamped); frequency–volume chart focusing on nocturnal urine volume and nocturnal voids; serum/plasma copeptin (± serial), serum/urine osmolality; urine sodium/urea excretion; exploratory urinary AQP2 measures (e.g., EV-AQP2 where feasible)	Pragmatic warming strategies in winter: passive pre-bedtime heating, thermoneutral bedroom temperature (heating/insulation/heated bedding), and exposure-fidelity monitoring with sensors; in trials, standardize evening fluid/sodium to reduce confounding	[16,17,18,19,20,26,27]

Notes: Each row includes a Pop/Stage tag indicating the primary evidence population and menopausal-stage specificity. Pop/Stage tag legend: T = transition stage-stratified (STRAW + 10); M = midlife age-window (~40–60) without staging; G = general adult/non-stage-specific human; O = older adult; X = animal/ex vivo/in vitro; “+” indicates mixed evidence. Evidence tags (L1–L5) are defined in Supplementary Table S1. Rows lacking transition-stage-specific evidence are included as a mechanism-to-measurement roadmap and should be interpreted as extrapolated (hypothesis-generating), not as established transition-specific associations. Abbreviations: AQP2, aquaporin-2; AVP, arginine vasopressin; LUTS, lower urinary tract symptoms; OAB, overactive bladder; TRPM8, transient receptor potential melastatin 8; V2R, vasopressin V2 receptor.

## Data Availability

This article is a narrative review based exclusively on previously published literature. No new data were created or analyzed; therefore, data sharing is not applicable.

## Supplementary Materials

The following supporting information can be downloaded at: https://www.mdpi.com/article/10.3390/nu18050825/s1, Table S1: Representative database search strategy and qualitative evidence-grading rubric used in this structured narrative review; Table S2: Approximate caffeine content in common beverages and foods (typical serving sizes).

## Abbreviations

The following abbreviations are used in this manuscript:

A1	Adenosine A1 receptor
AQP2	Aquaporin-2
AVP	Arginine vasopressin
CBT	Cognitive behavioral therapy
CYP1A2	Cytochrome P450 1A2
EV-AQP2	Extracellular vesicle aquaporin-2
FVC	Frequency–volume chart
L1	Human intervention
L2	Human observational
L3	Mechanistic physiology
L4	Animal/ex vivo or in vitro models
L5	Hypothesis-only/integrative inference
LUTSs	Lower urinary tract symptoms
OAB	Overactive bladder
STRAW + 10	Stages of Reproductive Aging Workshop +10
TRPM8	Transient receptor potential melastatin 8
V2R	Vasopressin V2 receptor
UI	Urinary incontinence
