# Supplementary material for: Dietary Caffeine, Cold Exposure, and the Estrogen–TRPM8 Axis: A Nutri-Environmental Model for Lower Urinary Tract Symptoms in the Menopause Transition: A Narrative Review
Source: Nutrients. 2026 Mar 3;18(5):825. doi: 10.3390/nu18050825 (PMC12986663; doi:10.3390/nu18050825)
Supplement: Supplementary file 1 [file nutrients-18-00825-s001.zip › nutrients-4158686-supplementary.pdf]

Supplementary Materials

Nutrients narrative review (submission)

Manuscript title: Dietary Caffeine, Cold Exposure, and the Estrogen–TRPM8 Axis: A Nutri-Environmental Model for Lower Urinary Tract Symptoms in the Menopause Transition: A Narrative Review

Authors: Dong Hee Lee; Jeong Jun Park (corresponding author)

Correspondence: jeongjun.park@cha.ac.kr

The following supporting information is provided to improve transparency and practical implementation: Supplementary Table S1. Representative database search strategy and qualitative evidence-grading rubric used in this structured narrative review; Supplementary Table S2. Approximate caffeine content in common beverages and foods (typical serving sizes).

Supplementary Table S1. Representative database search strategy and qualitative evidence-grading rubric used in this structured narrative review.

Component	Details
Databases searched	PubMed, Embase, Web of Science; citation tracking of key articles.
Coverage window	From inception through January 2026 (last search run: January 2026).
Core concepts combined	Menopause transition/perimenopause; LUTS/nocturia/nocturnal polyuria/OAB; caffeine/coffee; cold exposure/seasonality/indoor temperature/passive heating; vasopressin/copeptin/AQP2/V2R; TRPM8; sleep/falls.
Example PubMed query (illustrative)	(menopause transition OR perimenopause OR "STRAW" OR midlife women) AND (nocturia OR "nocturnal polyuria" OR "overactive bladder" OR urgency OR frequency) AND (caffeine OR coffee OR paraxanthine) AND (cold OR season* OR "indoor temperature" OR "passive heating") AND (vasopressin OR copeptin OR aquaporin-2 OR V2 receptor OR TRPM8).
Study prioritization	Peer-reviewed human studies with urinary outcomes in midlife women (operationally defined as approximately 40–60 years and/or studies reporting STRAW + 10 stages when available) and, when available, menopausal stage information; supplemented by older-adult or non-stage-specific human studies when midlife/stage-stratified data were sparse, and by mechanistic physiology/translational models to inform hypothesis-generating inference. Where menopause-stage-specific epidemiologic/clinical evidence was unavailable, mechanistic links were treated as extrapolated and explicitly labeled using evidence tags (L4–L5).
Qualitative evidence tag (recommended for tables/figures)	These tags were used to transparently denote the evidence type (L1–L5) and the primary population/stage applicability (Pop/Stage) of mechanisms summarized in the tables/figures. L1: human intervention (randomized, crossover, or controlled trial); L2: human observational (cohort/case–control/cross-sectional); L3: mechanistic physiology (human or integrative physiology without clinical endpoints); L4: animal/ex vivo or in vitro models; L5: hypothesis-only/integrative

	inference (extrapolated mechanisms) proposed for future testing.
Pop/Stage tag (recommended for tables/figures)	T = transition stage-stratified (STRAW + 10); M = midlife age-window (~40–60) without staging; G = general adult/non–stage-specific human; O = older adult; X = animal/ex vivo/in vitro. Use “+” when evidence is mixed.

Supplementary Table S2. Approximate caffeine content in common beverages and foods (typical serving sizes).

Source	Typical serving	Approximate caffeine (mg)	Notes (variability / guidance)
Brewed coffee (drip/filter)	240 mL (8 oz)	~80–120	Highly variable by bean, roast, and brew strength; use product/brew-specific estimates when available.
Espresso	30 mL (1 oz)	~60–80	Single-shot values vary; espresso-based drinks may include multiple shots.
Black tea	240 mL (8 oz)	~30–60	Steeping time and tea type substantially affect content.
Green tea	240 mL (8 oz)	~20–45	Matcha preparations can be higher; variability is common.
Energy drink	250 mL (typical can)	~80–150	Check label; some products are higher per serving and may include additional stimulants.
Cola-type soft drink	355 mL (12 oz)	~30–50	Brand-dependent; 'diet' versions are similar for caffeine.
Dark chocolate	30 g (1 oz)	~10–30	Cocoa percentage matters; milk chocolate is usually lower.
Decaffeinated coffee	240 mL (8 oz)	~2–15	Not caffeine-free; content depends on process and brew.

Note: Values are approximate ranges intended for dose-translation and taper planning; caffeine content varies widely by product and preparation. Whenever possible, use manufacturer labels and standardized serving definitions in research protocols.
